# Think Twice: First for Tech, Then for Ed

**DOI:** 10.1007/s42979-022-01538-7

**Published:** 2022-12-26

**Authors:** Panos Photopoulos, Dimos Triantis

**Affiliations:** Department of Electrical and Electronic Engineering, University of West Attica, Athens, Greece

**Keywords:** Technology driven change, COVID-19, Blended learning, Online learning, Face-to-face education, Technological determinism

## Abstract

The embodiment of technology in education can make learning easier, more enjoyable, and more accessible. From Learning Machines to artificial intelligence (AI), educational technology has repeatedly tested its strength as an aider or a substitute to in-person teaching. During the COVID-19 pandemic international organisations promoted the idea of the transformation of education using technology. Comparison of their texts published in 2020 with texts published in 2021 indicates that much of the early enthusiasm concerning the transition from in-person to remote learning gave its position to more thoughtful accounts after considering the learning losses and students’ disappointment from the disruption of in-person relationships. This publication highlights aspects of education technology usually overlooked in futuristic accounts of education. Adopting a non-deterministic view of technology attempts to contribute to the more human-centred incorporation of technologies in education.

## Introduction

The pandemic has pushed the world into the deepest global recession, with unprecedented negative consequences for most people. The crisis exacerbated pre-existing social inequalities and increased unemployment, poverty and insecurity. According to the International Labour Organization, job losses worldwide were equivalent to 255 million full-time jobs. The younger people were more severely affected, and youth employment decreased globally by 8.7% [1]. For the euro area, between 2019 and 2020, male unemployment increased by 8.0% and female by 6.6% [2]. In the US, the unemployment rate jumped in April 2020 “to a level not seen since the 1930s” [3]. The implications were severe for the people of poorer countries where the lockdown impacted the socioeconomically deprived families and the families of skilled workers [4].

The lockdown and the disruption of social relationships also had devastating implications for people's mental health, especially those packed in the building blocks of big cities. Research conducted among Greek University students revealed an increased rate of clinical depression, increased frequency of major and severe depression, and an increased number of suicidal thoughts [5]. Future projections are also disappointing. Each percentage point reduction in the global economy will result in a 10 million people plunged into extreme poverty worldwide [6, 7].

However, for the big corporations of the digital economy, the health crisis was an opportunity to expand their operations further and penetrate education. In this endeavour, they were not alone. International organisations, media, and politicians promoted the idea of a society moving forward with virtual schooling. The argument said that the health crisis revealed that traditional education had reached its limits and was inappropriate for the future. The transition from in-person to remote teaching was not a temporal solution but an intermediate step for the total transformation of education. The transformation rhetoric assumed that up to the coronavirus outbreak, online learning was an inaccessible type of education that students-parents and teachers would love after they tried it [8, 9].

After the pandemic broke out, past partnerships between international organisations, prestigious universities, and big corporations [10] expanded, and new ones were announced [11–14]. In April 2020, UNICEF [11] and Microsoft [12] announced that the “Learning Passport” platform had undergone rapid expansion to facilitate country-level curriculum for children whose schools closed due to COVID-19. In October 2020, Sony Corporation of America announced its participation in the same program [13]. A few days later, UNICEF announced that Learning Passport users from Jordan, Somalia, Timor-Leste, and Ukraine would have access to science content developed by Twigeducation [14] and the Imperial College [15].

Apart from the partnerships between universities and private companies under the auspices of international organisations, some universities searched for private partners to digitise their courses. During the first month of the COVID crisis, the online learning platform Coursera received enquiries from more than 6000 universities in 120 countries [16]. The technology vendors inundated with partnership proposals from public universities seized the opportunity to promote partnerships that would add value to their brand name. For the universities collecting fees, the response to the COVID crisis was also a question of positioning in the competitive market. For some universities going digital was considered an attractive strategy [16, 17].

Although the rapid approach necessary for Emergency Remote Teaching (ERT) raised concerns about the quality of the courses [18] delivered, it was the most practical solution. The university teachers balancing disappointment, frustration, and long working hours ensured educational continuity [19]. As the Guardian has written, history will record that teachers were better prepared than the government officials [20]. Technology had a central position in education transformation [21–25].

“Post-COVID education: A case of a technology-driven case?” [26], CSEDU 2021 attempted to identify elements regarding the future of education, collecting information from texts and publications published by international organisations in 2020 immediately after the COVID-19 outbreak. The narrative on post-COVID education emphasised the benefits of online learning. The publication noticed that the proposed education transformation would eventually displace the highly specialised professors and transfer their knowledge and expertise to platforms supported by technical and administrative staff. The digital paradigm would replace teaching with knowledge availability and students with isolated learners. A top-down transformation of education was considered a probable scenario for the near future.

The present publication enriches the information of our previous publication and reviews its findings. The outcomes of the 'massive global social experiment' [27] of remote learning have partly reshaped the narrative of the international organisations. However, the dominance of technological determinism hampers much of what is intellectually and practically crucial in thinking about technology and education. For rethinking education to be fruitful, we have to reconsider technology before education.

This publication collects information regarding the future of education as described in the texts of various international organisations. Quiet importantly, a gap was identified in the views expressed in 2020, immediately after the pandemic broke out, and those expressed in 2021. In the meantime, research showed the alarming learning losses that occurred during the pandemic. Besides, this publication attempts to revisit the rethink education proposal from a non-deterministic standpoint. The aims of the paper are: To detect the emerging view of the international organisations searching in documents they published immediately after the pandemic in 2020. To identify publications of the international organisations referring to the effects of the pandemic on students' learning. To record the extent to which the effects of the pandemic affected the emerging view of the international organisations. To reconsider the texts of the international organisations from a non-deterministic perspective.

## Rethink Education

The health pandemic coincided with enthusiasm for the total transformation of in-person education to online learning. International organisations, private companies, politicians and the media agreed that the experience of remote learning would create a demand for digital education. Some commentators were more careful and spoke of a massive experiment. Others took the idea of leadership to its extreme with expressions like “online universities are here to stay”, “there is no going back”, and “why future students will learn online” [26]. However, for the teachers who already had an experience with synchronous remote teaching, it was more than clear that this was going to be a challenging period [19], while the probability of learning losses would be high, no matter how hard they tried.

The international organisations showed particular interest in post-COVID education, adopting different approaches and making recommendations. More universalistic organisations like UN, UNICEF and UNESCO showed great concern for supporting the students, especially the poor and more vulnerable, and ensuring the continuity of education. On the one hand, they stressed the importance of in-person education, while on the other, they participated in networks with private companies to enhance the digital alternative. A UN document [22] praised the digital solutions for improving teaching and learning and stressed that they must be institutionalised in the aftermath of the pandemic. However, it warned that education could not depend on digital platforms controlled by private companies, to conclude that the solution lies in open access to digital educational resources. The World Bank based its texts on research findings, while it was more inclined towards blended learning as a suggestion for the future. The OECD steadily promoted the private sector's involvement in post-COVID education, promoting the idea of a complete transition to online learning. Finally, the World Economic Forum considered the idea of a total transformation of society from in-person to digital. A 'star trek' society where all transactions and activities will go digital and remote. One cannot assert a one-to-one correspondence between international organisations and proposals for post-COVID education. Various perspectives diffuse in their texts resulting in a broad picture that gives a message of a change to come.

International Organisations' texts concerning the future of education reflect a specific worldview. Critical discourse analysis questions the possibility of ideology-free statements considering that statements and texts unavoidably reflect a particular worldview, affecting how the readers perceive and give meaning to their experiences. In this sense, all texts construct subjectivities, maintain, and legitimise social inequalities [28]. OECD, for example, is an international organisation having a clear political and ideological commitment, and some of its documents serve the purpose of advising governments in the formulation of their policies [29]. In September 2020, OECD published the four scenarios for the future of education. These scenarios define the locus of the future of education compatible with OECD's values and world view, which are commitment to democratic governance and adherence to free-market principles [26].

The involvement of the private sector in education is central to OECD's scenarios [21]. In the first scenario (Schooling extended), the control of education typically lies in the hands of the governments, and attention focuses on the curriculum. International digital learning systems collaborate with public organisations. As learning providers search for economies of scale, course content is standardised across countries, ensuring uniformity of assessment and curriculum. Private education providers act internationally, sharing learning resources and collecting learning data from different countries. The teacher still has some role in organising the instruction, but there is great diversification of professional profiles in schools. Feedback to students and parents is instantaneous via platform applications. One may think of prestigious universities offering courses internationally. Students studying for a degree can choose several online courses from different universities and pay a certain fee. Private companies provide media design services, and data companies collect learning data from the international students to feed algorithms that decide upon delivering learning content for personalised learning. Government authorities play some role in decision making, e.g., deciding which providers to collaborate with, but their authority is undermined as their international partners gain power.

In the second scenario (Education outsourced), the curriculum has disappeared, and education results from individuals buying educational services deemed to be of value to the market. Flexibility in employment and unstructured education force the parents to get more involved in children's education. Since the curriculum is absent, prestigious schools are also rare, and the value of learning depends on how attractive it is to the employers. Therefore, employers involved themselves in education as learning providers. Private learning providers offer products to the learning market, while the state intervenes to provide benchmark and baseline assessment criteria to the learning clients.

The fourth scenario imagines a society that relies entirely on artificial intelligence (AI). The teaching profession has vanished, and privately-owned technological resources provide learning services (Learn-as-you-go). AI becomes the individual learner's assistant, suggesting learning patterns and connecting learners of similar interests. Formal and informal learning happens anytime, driven by the curiosity and interest of the individual learner. Global digital corporations play a central role as learning providers developing, maintaining, and powering learning systems, collecting and retrieving learning data. Boundaries between local and global have vanished, giving rise to a globally interconnected learning system.

In the third scenario, the school retains most of its functions. It is internationally informed but linked to the local community. The curriculum is influenced by local actors and facilitators or individuals interested in promoting education. School is an all-day activity, but learning is not confined to the classroom. It is based on diversity and experimentation.

Transformation of education is high on the agenda of organisations like UNESCO, the UN and UNICEF. In its announcement of the Global Education Coalition, UNESCO notices: “To be more resilient, equitable and inclusive, education systems must transform, leveraging technology to benefit all learners and building on the innovations and partnerships catalysed throughout this crisis.” [30]. UNICEF is more determined regarding education transformation in some of its texts: “The year 2020 has been a year of learning. And a year in which we discover that a new normal is not just possible; it is essential. There is no going back.” [31]

In August 2020, the UN [22] published 'Education during COVID-19 and beyond'. The document calls for expanding the right to education to include connectivity. It provides a rich picture of the consequences of the pandemic but no justification for the proposed recommendations. The Executive Summary, as well as the whole document, follows the steps identified by Davis's rhetorical analysis of persuasive texts [32] and derives its power from the careful navigation of the reader's emotions:

1. Identify a novel factor that affects modern life: “The COVID-19 pandemic has created the largest disruption of education systems in history.” 2. Contrast today's society affected by the factor with previous to highlight the importance of the factor: “The crisis is exacerbating pre-existing education disparities by reducing the opportunities for many of the most vulnerable children,” 3. Show how the factor has subverted aspects of life that people value: “Similarly, the education disruption has had, and will continue to have, substantial effects beyond education.” 4. Play with the audience's fears by pointing out the spread of this factor: “As fiscal pressures increase, and development assistance comes under strain, the financing of education could also face major challenges, exacerbating massive pre-COVID-19 education funding gaps.” 5. Show the way forward by suggesting ways to control the factor or at least live with it: “On the other hand, this crisis has stimulated innovation within the education sector… these changes have also highlighted that the promising future of learning, and the accelerated changes in modes of delivering quality education, cannot be separated from the imperative of leaving no one behind.”

## Learning Losses

Immediately after the pandemic breakout, academics researched the attitudes and feelings of the students during ERT. The research findings differed between regional areas, cultures, and countries, but some evidence indicates that the students were disappointed because of the lack of face-to-face communication with their colleagues and teachers [33]. In 2021, there was enough evidence that distance education during the pandemic resulted in learning losses in poor and developed countries.

However, some surveys provided convincing evidence of learning losses and students' frustration during the period of remote learning. A survey conducted by European Students' Union in April 2020 [34] revealed students' clear preference for in-person interactions between students and teachers. The participants valued communication with their teachers as a source of inspiration and the experiences they obtained from social life on campus. A World Bank publication [35] found that school closures resulted in significant learning glasses despite teachers' efforts. The publication sites early results from research conducted in developed countries (Belgium, Netherlands, Switzerland and UK) which confirmed learning losses and increases in learning inequalities.

An OECD publication [36] concluded that, for a significant proportion of pupils, learning during school closures was almost non-existent. The learning opportunities were significantly reduced, and the negative consequences of the school closures were higher for children coming from disadvantaged families. Research conducted in Germany found that the students spent half of their time on school-related activities compared to the pre-COVID period. Instead of studying, they spent more time on TV, mobile phones and playing computer games (5.2 h/day), while low achieving students replaced learning with passive activities. In conclusion, students' learning during 2020 was significantly less than in 2019. The learning losses humper the acquisition of new skills and undermine some skills the students already had. According to estimations from research in the economics of education, each additional year of schooling increases life income by an average of 7.5–10%. At a national level, the learning losses will decelerate economic growth. [36].

The impact of the learning losses alarmed UNESCO [37] because they put the targets of the Sustainable Development Goal 4 in danger. The implications of school closures on learning delayed the achievement of the DG 4 intermediate targets and increased the cost of its implementation. The school closures during 2020 were equivalent to 1/3 of a school year's learning. According to historical data, the learning losses will lower the national GDP by 1.5% over the century [36].

Apart from the impact on learning, school closures negatively affected students' mental health and social skills. As schools reopened, reports from teachers and educators revealed behavioural problems, inability to focus during classes, violence [38], difficulties in communicating with fellow students and teachers [39] and increased cases of misbehaviour [40]. The World Economic Forum insisted on young people's severe mental health deterioration during the pandemic [41].

## Rethink Education Reconsidered

Crises create shock, which lowers the psychological barrier to bold interventions. Change leaders use a well-crafted rhetoric to convince their followers to accept changes. Distance learning and distance working during the pandemic fuelled imaginative accounts of a digitalised life. The health crisis was the excuse for the proposed transition to digital human interaction, e-commerce, online education, and remote work for the World Economic Forum [41]. “Rethink education” and “rethink work” were promoted using similar rhetoric schemes [32]: “It is time to rethink where, how and why we work” [42]. Forbes considered working “Beyond The Pandemic: There is no going back to the old ways of working… it would be naive to think everything can—or should—return to “normal” after the pandemic ends. It's clear that work from home is not going to go away” [43]. The digital edition of the TIME showed the way to the future: “It's Time to Radically Rethink How We Work” [44].

Activities that traditionally belonged to the realm of face-to-face interaction went remote. Learning to live and perform from a distance during schooling can be an asset in tomorrow's distance working environment. Additionally, if education aims to prepare tomorrow's workforce, then a schooling system adapted to minimum human interaction sets out the cultural basis for accepting a workplace with similar characteristics. The proposal of online learning derives its force not only from its pedagogy but also from its potential to familiarise students with working under minimum human interaction.

 Remote learning during the pandemic generated learning losses [45], which decelerated the achievement of the DG 4 targets [37], undermining economic growth. The exacerbation of inequalities and worries concerning students' mental health [41] indicated that the rapid top-down transformation of education had to wait.

In July 2021, the Director for OECD's Directorate of Education and Skills announced that digitalisation must not replace student–teacher and student–student relationships. The transformation of Higher Education was still ahead and the primary role for its realisation passed to the universities. The universities will have to “commit to the development of a next-generation learning environment”. This implies large-scale investments in hardware and software, sufficient time and training for teaching staff, and adopting pedagogical and assessment approaches suitable to the new digital environment [34]. Regarding primary and secondary education, another OECD document [46] explained that the strategy to increase the use of digital technology up to 2025 would include training the teachers and developing digital learning tools designed for young children. The strategy for the digitalisation of education is still valid; however, the abrupt change suggested in the 2020 OECD documents is replaced by a gradual one. Incremental changes proceed through the training, institutional policies, and interactions with the material features [47] of technology to alter the attitudes of the change recipients.

The research findings concerning the implications of remote teaching, school closures and movement restrictions sensitised the international organisations to reconsider their position regarding rethinking education. UNESCO, in July 2021, criticised the governments for shutting down schools and keeping them closed for prolonged periods, even when the epidemiological situation did not warrant it [48]. The publications of the World Bank encouraged blended learning and the cultivation of a culture of gathering rigorous data on student outcomes, expenses and performance to inform data-driven decision making [49]. The World Economic Forum adopted a holistic view where digital technologies will become an accepted, “must-have” part of the blended working and learning environment [27].

For international organisations, the digitalisation of education is the answer to developing the human capital to serve economic growth and assure equal access to education for all. They are among the driving forces of the transformation proposal shaping the global vision of education, building robust networks and acting as advisors of the governments [50–52]. One can identify several factors that influence the future of Higher Education. These include:

1. The budget cuts since 2008 [26] and the increasing student debt in the US [17]; 2. The Tayloristic imperative of doing more with less. Online learning is praised for its pedagogy and cost-effectiveness [26]. However, developing high-quality online courses is expensive, and cost-effectiveness for the consumer results only in cases when the volume of sales is large, i.e. whenever there are economies of scale. Online learning requires a global market [21, 51] and strict management of the operations. In that respect, OECD, with its International comparisons (PISA), has paved the way for a global education market [26]; 3. The historical interest of private capital [53] for expanding its activities in education to realise profits [54] and create new markets, e.g. data trading [55, 56]. OECD and the World Economic Forum consistently encourage the involvement of the private sector in education and the establishment of public–private partnerships [21, 41]; 4. The continuous effort of management to increase its control in universities and extract power from the teachers in both teaching and research. Data collected during online learning will put the entire operations under management’s decisions, from curriculum decisions to research agendas [55]; 5. The belief that technology is an inherently powerful, positive, and autonomous force that generates material comfort for the world.

## Technology and Progress

For nearly a century, education has gone through changes [57], but classroom education has, up to now, stood the test. However, the digitalisation of Higher Education differs considerably from past attempts to reform universities because of the influence its proponents exert on society. These include international organisations and big corporations interested in investing in the education market. Education technology has a positive meaning and is considered synonymous with progress. Technology seemingly generates economic growth and greater equality, provided everyone has access to it [58].

Technology seems to result from a process similar to natural selection. Successful technologies result from human efforts to push boundaries back and move forward. The alternative technologies are competitively evaluated on the basis of their technical merits. The technology that survives this competition is the best technology available regarding its technical superiority and economic efficiency. It is a kind of technological Darwinism in which prevailing technologies have passed a sequence of filtering mechanisms [59].

While Western societies consider technology synonymous with progress, several societies have rejected powerful technologies in the past. Communities made self-conscious technological choices and rejected technologies they already used. Examples are the wheel in North Africa, the shotguns in Japan and the metallurgy of gold around the Caucasus area. Technological determinism seems to require a free market. It is a conception characterising individualistic societies that embrace laissez-faire economics [60]. Maxine Berg [61] identified the origins of technological determinism in the early analyses of political economists during the nineteenth century. The newly emerged political economy wanted to calm the frustration of the working classes from the disruption of their lives caused by the introduction of technology. They connected the abstract ideal of economic development they had invented to their perception of the advancement of technology. This spur relationship was never seriously challenged by the critics of political economy and survived to our days. However, its propelling force has exceeded its limits.

The recent technological developments are strikingly different compared to their predecessors. Modern technology and automation displace labour without increasing productivity, which means less employment in the future. The standard approach suggests that when technologies increase productivity, they also increase labour demand. However, AI affects negatively the employment of the highly skilled workforce. Thus, the possibility of counterbalancing unemployment by training [62] is not an option. In OECD scenarios, ICT and AI destabilise employment relations in education to generate economies of scale. They change the nature of teachers’ work, introduce a diversity of professional profiles and flexible working arrangements, challenge faculty’s status, and eventually, the teaching profession vanishes. However, disrupting university employment in a fully automated economy has wider negative consequences. The displacement of professors will devastate the local economies since privately owned companies have a much lower marginal propensity to consume than those losing incomes and jobs [63]. Therefore, the relationship between technology and progress is challenged as Higher Education delves into the fourth industrial revolution.

This section challenges commonly held beliefs of technology as an inherently positive project and its adoption as equivalent to progress. Besides, the evaluation of technological solutions is unavoidably related to its repercussions on the individual. The learning losses [45] occurred during the school closures, and the behavioural problems recorded after returning to schools [38–40] challenge the a priori goodness of learning disjunct from face-to-face sociality. Accepting technology on the basis of its performance alone, as the Technology Acceptance Model suggests [64], makes technology an authoritarian instrument of control, negating the joy of in-person communication or the theatricality of the classroom.

## Technology as an Independent Entity that Brings Changes in Education

Deterministic views consider technology as an independent entity that determines the course of our society. The deterministic interpretation of technology was manifested in the media and the web pages of international organizations during the pandemic. In some texts, technology defined direction in time and space. The transition from face-to-face to distance learning was the future and a step forward. In-person teaching was the past and a step backwards. Here are some examples: NYT: “The Future of College Is Online, and It's Cheaper” [65], The Guardian: “We shouldn't go back to lectures: why future students will learn online” [66]. In other cases, technology was the independent factor in transforming education: “How technology will transform learning in the COVID-19 era” [67], “How Technology Is Changing the Future of Higher Education”, and “There is no going back” [26]. Technology was an abstract entity generating change. In that perspective, changes are not the result of human decisions; technology necessitates them. Since technology is the “future,” these changes are manifestations of progress, so they must be accepted. Understanding technology as an autonomous entity that controls the course of our society neutralizes our critical faculties. It exposes us to the feeling that our collective life is uncontrollable [68].

Management scholars comment that the employees, i.e. those experiencing organisational changes, remain inarticulate concerning their reactions during change management because managerial action renders them incapable of action. Well designed and carefully orchestrated initiatives assisted by blind faith in technology paralyse rational thinking. As Weick has noticed: “shy people find it difficult to take action, alienated people find it difficult to sustain action, and depressed people find it difficult to do both” [69].

Contrary to the misconception that technology changes organisations or education, reality says that an agenda for organisational changes accompany most new technology implementations in businesses. As Leonardi [47] argues, management takes advantage of the material features of technology to alter communication patterns and thereby affect the sociality of the organisation. Moreover, in management's rhetoric, technology becomes the “objective force” that legitimises changes in employees' perception of themselves and their relationship with the organisation. In this rhetoric, technology, not management's prerogative, causes lay-offs, intensifies work, reduces wages, and calls the employees to perceive their objectives as parallel to the “survival of the organisation”. Following the steps identified by Davies [32], management rhetoric plays with the fears of the employees to affect the meaning of work, employee's values and identity. The co-occurrence of organisational change with technology introduction is mistakenly understood as a causal relationship where technology causes organisational changes. It is instead the other way around. The purposeful action of management uses properly designed technology as an excuse to impose changes on the shop floor.

The persistence in the universities' digitalisation also relates to agendas of employment changes, aiming to increase managerial control over the faculty. A comparison of the 2001 and 2020 OECD scenarios highlights this direction. The 2001 Scenario 1 refers to the “Dominance of the classroom/individual teacher model.” Twenty years later, this has been replaced by “the classroom/individual adult model” [21]. As the technological arrangements capture the teachers' intelligence, their role becomes complementary to learning [70]. The teachers' knowledge, skills, and culture are subsumed into the technological artefact [71]. Teachers' didactic and pedagogic role is of no interest to the 2020 OECD scenario. The traditional managerial dictum “fit the man to the job” becomes fit the teacher to technology by training. As the universities are pushed to operate more efficiently, the autonomy of the academics to perform administrative, research and teaching tasks is reduced [72].

Although the texts of the international organisations reflect the optimistic view that technology can solve old existing problems in education, Sarewitz and Nelson [73] argued that technology cannot fix problems in education. Their analysis compared two technological artefacts that became popular during the pandemic: vaccines and education technology. For technology to work as a technical fix, it must fulfil three conditions:

First, for technological fixes to work, they must largely embody the cause-effect relationship connecting the problem to the solution. A vaccine's effectiveness does not depend on the person who gives or receives it, nor on the characteristics of the healthcare system. Vaccines protect people even within dysfunctional health care systems. The vaccines are effective because their technology embodies the relationship between the cause (vaccination) and effect (protection). Unlike vaccines, books, software, the internet, and communication technologies do not provide the “basic go” of learning. Other factors, e.g. student engagement, may screen the effectiveness of technology in knowledge acquisition. Research showed that, during the COVID crisis, learning losses were higher among students who received less support from their families. The problem was more prominent for students from socioeconomically disadvantaged families and less educated parents [36]. The findings reveal that socioeconomic status, cultural values, personality and technology influence learning. Decades of research have not identified a single factor that unavoidably would improve education. Therefore, a relationship between cause (education technology) and effect (learning) does not exist.

Second, a set of unambiguous criteria must exist to assess the effectiveness of the technological fix. Although one may oppose vaccines on moral grounds, their role in protecting public health is hard to argue against. On the contrary, in education, such unambiguous criteria do not exist. Let us see how this point unfolds using an example from the pandemic. A 2020 survey among European university students revealed a clear preference for face-to-face teacher-student interaction. According to OECD, “To meet the expectations of these learners, higher education institutions will need to create learning environments in which digitalisation expands and complements, but does not replace, student–teacher and student–student relationships.” [34]. However, similar results from a US study were attributed to the students' poor online learning experiences during the pandemic [74]. The absence of clear criteria in judging the effectiveness of ERT resulted in some kind of epistemic relativism where similar evidence supported opposing theories. Although the evidence is worth trusting, there is no agreement on its interpretation.

Third, successful technological solutions would result from an existing standardised technical core, like the vaccines developed against the coronavirus. However, such a technological core does not exist in education technology. Various technological innovations have been introduced in education and challenged in-person teaching throughout the years. TV education, videoconferencing, and computer-assisted instruction attracted academics' enthusiasm. Soon after their introduction, they lost their propelling power without leaving behind some technical core for developing a technological fix in education.

Online learning does not fulfil the conditions set by Sarewitz and Nelson for technological fixes to work. Therefore, its success will be conditional and dependent on the learner's characteristics and the specificities of the learning environment. The anticipation that online learning will solve old existing problems is ill-founded and risky in a world of limited resources.

## Fifty-Five-Year-Old Novelties

Today's technology resulted from human efforts over hundreds of years to understand how things work and make valuable artefacts. A technological product's historical and social aspect is not apparent when we use it. When the historical, social, cultural, and economic aspects of an artefact are unknown, it becomes a mystical object. We feel comfortable with its properties and usage, although we do not understand its nature. It acquires “phantom-objectivity” [68].

The history of artefacts and ideas in education is helpful for their evaluation. The current enthusiasm over individualised learning (I.L.) creates the impression that I.L became possible only recently because of the advancement of ICT. However, I.L. is discussed in publications of the seventies, while historical research identified its origin in the late nineteenth century [53]. Patrick Suppes, in 1966, proposed I.L. as a particular type of instruction that would make the learning experience unique for every student [75]. I.L. was a desirable feature of education, independently of the technology involved [76]. The introduction of the early computer systems in the universities generated interest in individualised education during in-person classes. However, computers and the aim of individualised learning did not affect the teacher's identity. It was considered that “automation can be introduced in individualised education as a means of assisting the teacher” [76]. Computer-Assisted Instruction (CAI) was closely related to I.L. without affecting the existing relationships. It was supportive of education, improving the work rates of the students [77] without affecting student–teacher and student–student relationships [78]. Computer-assisted instruction was used to increase students' attention during classes while it could adjust instruction to students' rate [77]. Other publications suggested more sophisticated employment of computers to help the students program their learning [78].

However, the Fordist view of technology was also present, i.e., the idea that computer systems could substitute for the teacher. For example, specific computer programs were introduced to supplement the shortage of mathematicians in England [79]. At the same time, there were claims that specific systems can effectively simulate the action of the human tutor [79]. As it happens today, several enthusiastic scholars assumed that computer systems “can be expected to play a major role in transforming the educational process” [78].

The interest in data collection was the predecessor of today's learning analytics. Learning data collection and statistical treatment were meant to enhance the learning experience [76] and adapt learning to each student's needs [78]. The computer collected the learning data, and a decision-making system would recommend more suitable learning paths for each student [78].

All the feasible technological characteristics of online education are present in embryonic form in publications of the early years of computers in education. These include: individualized learning [75–77], learning analytics [76, 78], using technology to provide education for the disadvantaged [78], involvement of “commercial companies” [79], using computers to facilitate life-long learning [78], and the constant enthusiasm that technology will bring revolutionary changes in education [78].

Techno-optimism has been continuously present in education research. In 1990, multimedia and computers developed a widespread optimism that global computer networks would make distance irrelevant for course delivery [80]. As it happens today with online learning, the researchers compared students' performance in face-to-face and remote settings to conclude no significant difference [80, 81]. Videos were considered superior to video conferencing because they allowed the students to pause and rewind [82]. Combining technologies would allow the teacher to gather information and determine the bored students and those having questions, thereby modifying and personalising their learning experience [83]. Around 2000, techno enthusiasm was centred around massive open online courses, but in-person teaching proved its longevity [17] again.

## The Political Qualities of Technology

Once provocative, the idea that technology is political attracts now increasing attention. When the positive relationship between technological innovation and democratic progress is challenged [84], the question of the political qualities of technology becomes equally crucial to technological innovation itself. In that respect, the Ford Foundation hired technologists to understand better the growing influence of data and technology on social justice issues [85]. Technology has different effects on different groups of people. It generates arrangements of power and authority in the associations between people, which influence their activities within these arrangements [86–88]. For example, in the US, during the pandemic, the digital platforms allowed full-time university employees to continue working. However, 570,000 people providing administrative support, food services or teaching under contract lost their jobs during the “greatest job losses on record” [89]. Additionally, platform technology, a transformative force in university education [34], has been related to learning losses during school closures [90].

The discussion on the political qualities of technology examines what people do with technology and what technology is doing with us as it becomes embedded in the socio-material reality we live in [87]. Focusing only on the first aspect, i.e. what we can do with technology, results in ill-founded enthusiasm for technological fixes to long-standing problems. However, “It is an illusion to think that online learning is the way forward for all” [91].

## Political by Design

For Langdon Winner [86], technologies are ways of building order in our society. As he notices, some technologies are inherently political [87]. The introduction of technology at the workplace is inextricably related to the central managerial concerns of efficiency in operation and power relations between the workers and the management. The history of technology shows that technology introduced in the name of more efficient operation also altered the social relationships and shifted the power balance in favour of the management. Ford motor is a historical example of a successful technology introduction with repercussions for society. Ford’s managers and engineers transferred the skills from the skilled worker to sophisticated and complicated machines operated by unskilled labour [93]. The moving assembly line defeated the workers on the shop floor, diluted skills, degraded work, and completely changed the social relations between the workers as well as between the workers and the managers [92]. There are also cases of technologies introduced to ensure managerial control and authority without aiming to improve the efficiency of the operations [92]. David Noble has shown that numerical control prevailed over record-payback technology for reasons related to managerial authority alone [59].

Up to 2000, the academic teachers were among the few to maintain control over their work. Direct supervision could work only in cases of gross negligence. Standardisation of the work possess was difficult because of the complexity of the work process. Standardisation of outputs was the only applicable control mechanism. However, the complexity of educating people granted complete control of operations to the teachers. Traditional control mechanisms were ineffective, which caused frustration to governments and management [94]. Management was eagerly seeking ways to control the operators of the professional bureaucracies, including academic teachers and took advantage of the lessons learned from quality management.

Under this framework, the students are customers and what organisations should strive for is customer satisfaction [95]. This led to quality assurance processes, including student evaluations of the faculty. The management followed Taylor's good-old recommendation of management as the missionary fighting “soldiering”. The identity of the professors as the individuals who take full responsibility for their work was challenged, and management asked the students-customers to give feedback on the faculty's performance [96]. Did student evaluations improve teaching? Research has shown that the students' evaluations of the faculty improve teaching in rare cases under specific conditions [97]. The students have realised that the process is nothing more than a bureaucratic exercise. [98] Although, in principle, they like the idea of continuous improvement via evaluations, their participation is low. Still, management emphasises students' evaluations of the faculty as a tool to undermine teachers' authority and control over their work.

The digitalisation of the university linked to the promise of profit creation and economies of scale implies particular choices regarding technology. The upsurge of managerial control after 2000 affects the design of education technology. Quality teaching is inherently linked to the individual teacher. For economies of scale to accrue, management must deploy technological artefacts that dissociate the teacher's intelligence from teaching. Teachers' intelligence, videos, tests and intelligent tutoring are bundled to create a new product. Finally, strict operations management ensures operational efficiency. The digital transformation of Higher Education takes managerial control to its extreme subsuming teacher's intelligence to management authority.

The power balance between employees and management is central to decisions related to technology. Over-reliance on technology will affect teaching, dilute skills, degrade work and transform social relationships in schools and universities. Technical and organisational innovation will displace academic expertise, and administrative staff will supervise the knowledge delivery system run by computers, virtual classes, and networks. Technology will standardise the design and the content of the learning product. ICT using the most recent advantages of information technology, will transfer academic knowledge and skills from the teachers to sophisticated and complicated technologies [99]. In effect, this will Taylorise education and reduce its cost. Some teachers will play the role managers and engineers played in Ford's lines: set up the learning machine. This will bring new forms of control to schools and University departments [26, 93].

Another interesting aspect of education technology is the deployment of artificial intelligence (AI). AI is a platform technology which can develop in various directions as production or commercial technology. This is not new in technologies, but development direction is essential for AI. Up to now, AI has developed to substitute labour without increasing productivity, reducing the overall demand for labour. Mainstream optimistic views predicting that job losses in one sector will increase employment in another are not valid in the era of AI. “If all we do is continue down the path of automation, with no counterbalancing innovations to generate new tasks, the implications for labour are depressing” [100]. Whether AI in education will be substitutive or enabling is a matter of struggle that may impact technology design. If education technology focuses on generating economies of scale as OECD's scenarios suggest, employment in universities will decrease, and Edu businesses' short-term profits will increase. Analysis of job market data has shown that between 1999 and 2009, employment increased among the low-skilled labour only [62].

## Authoritarian Technologies

Apart from its design, technology is political because it does more than serve its practical purpose. Technology in society obtains political character, democratic or authoritarian. Dispersed solar systems are decentralized both technically and politically. Technically speaking, solar systems can be built at different places without the need for massive supporting infrastructure. They can be further expanded and cover the needs of local communities. Politically, because of their small scale and locality, they are negotiable and comprehensible in their role and operation without disturbing existing social relationships. On the other hand, nuclear power plants imply a techno-scientific-industrial-military elite simply because “without these people in charge, you could not have nuclear power.” [86]. The adoption of certain technologies unavoidably sets a number of conditions regarding human relationships. This distinction between technologies offers a framework for considering the techno-political status of various disjunctive technologies.

Classroom teaching is spatially and temporally defined. In-person teaching includes chalk and board, a projector, and computers with relevant material, i.e., a technology accessible, comprehensible, flexible, and controllable by its users. Because of its simplicity, technology allows individuals to evaluate the results of its use and make recommendations for improvement. School students, or their parents, can evaluate how the specific teacher takes advantage of the technology to ensure quality learning. In-person teaching technology allows much flexibility. The students can raise questions and co-produce the content of the lecture. In-person teaching is compatible with add-ons and modifications by its users. The flexibility of the teacher-centred model is the hidden variable that made the continuation of teaching during the pandemic possible. The simple technological arrangement of in-person teaching allowed adaptations in the emergent situation of the COVID crisis. During the pandemic, complex socio-technical systems, i.e. the universities, were able to adapt and secure continuity of education [101]. Does a complex technological system allow flexibility? Could online schooling continue “if communications infrastructures are targeted during a crisis with civil unrest” [102]?

Online learning involves a complex and spatially distributed technology unreachable and incomprehensible to its users. Online Learning is not only estranging but also alienating. The students have no say in the curriculum as they operate at the fringes of a complex system. They contribute their learning data, but they do not have any room to influence the operations. The curriculum and the content of each session are out of students’ influence or control. Students can only give thumbs up or thumbs down. However, criticism or praise is just information to the system interpreted under the system’s assumptions. Proper handling of learning data can prevent risks related to online learning technology but it cannot alter its political character.

The inherently authoritarian character of the technology allows its administrators to have complete control of the usage of learning data. Private companies offering education services will have the opportunity to use the learning data to advance their services and develop theories on learning. The professors will be depleted not only from teaching but also from their role as researchers [103].

## Conclusions

The COVID-19 pandemic reinforced the dominant narrative concerning the digital transformation of education, promoted by international organizations, politicians, and the media. This narrative assumes an a-priori positive role of technology in education. Remote education has helped millions of people to get a degree or certificate and peak a career. It would be difficult for someone to argue that online learning is not a more attractive and effective way of learning compared to correspondence education. For such reasons, online learning has an honourable position in the palette of non-traditional education. It is an attractive solution to those looking for a second opportunity in education, and under certain conditions, for marginalized and displaced people.

The pandemic showed that rapid changes in education are risky. Albeit the early enthusiasm for online learning, considerable learning losses occurred during the pandemic, delaying the implementation of the targets of the Sustainable Development Goal 4. There are worries concerning the mental health of the students and a strong preference for in-person relationships with peers and teachers. These findings alarmed the international organizations and postponed the option of an immediate transformation of education.

According to the OECD, the universities have to invest in hardware and software, training and adoption of pedagogical and assessment methods to allow the academic community to interact with the material aspect of online learning and prepare for a change in the future.

The lessons learned during the pandemic show that overenthusiasm with technology undermines the future of education. Rhetoric schemes like “there is no way back”, “the future is online,” etc., are meaningless outside the deterministic perspective of technology. Reconsidering the question of education technology in the light of its political qualities will revitalize the dialogue on the future of education and lead to more thoughtful decisions. Technology decisions are costly, and every investment must be considered carefully in a world of limited resources. Before we rethink education, we must first reconsider technology not as a hegemonic power determining our destiny but as the achievement of our collective effort.
